# Oxidative stress in *Physella acuta*: An integrative response of exposure to water from two rivers of Atlantic Mexican slope

**DOI:** 10.3389/fphys.2022.932537

**Published:** 2022-09-02

**Authors:** Jacinto Elías Sedeño-Díaz, Eugenia López-López

**Affiliations:** ^1^ Coordinación Politécnica para la Sustentabilidad, Instituto Politécnico Nacional, Ciudad de México, Mexico; ^2^ Laboratorio de Evaluación de la Salud de los Ecosistemas Acuáticos, Escuela Nacional de Ciencias Biológicas, Instituto Politécnico Nacional, Ciudad de México, Mexico

**Keywords:** lipid peroxidation, antioxidant system, freshwater mollusks, sentinel species, oxidative stress responses

## Abstract

Freshwater pollution is a complex mixture of xenobiotics due to the wastewater and the various chemicals routinely applied to agricultural lands that are discharged into water bodies. Xenobiotics can exert damage to the aquatic biota threatening the biodiversity of aquatic ecosystems. The oxidative damage and antioxidant responses have been widely investigated in freshwater organisms, mainly in fish and some invertebrates but in freshwater snails are scarce. This study aimed to assess the oxidative stress exerted by potential toxicity of water from two rivers of the Mexican Atlantic Slope (Tecolutla and Tuxpan rivers) in a freshwater mollusk *Physella acuta.* Lipid peroxidation level and a battery of antioxidant enzymes (Superoxide dismutase, Catalase and Glutathione peroxidase) were measured in *P. acuta*. The results are contextualized from an ecological point of view, associating the bioassay results with water quality characteristics. Water samples were obtained from three study sites for each river (in two seasons: Northern wind and dry). Twelve water quality variables were analyzed, and an additional water sample was used to perform a static bioassay for 96 h with snails grown in laboratory. After the exposure, we assessed lipid peroxidation level and the antioxidant responses of *P. acuta* exposed to water of rivers, and the Integrated Biomarker Response was computed. The highest lipid peroxidation level occurred in organisms exposed to water during the Northern wind season in both rivers. During this season, in the Tecolutla river, the superoxide dismutase activity was able to counteract the lipid peroxidation process, representing an adaptive response. In contrast, in the Tuxpan river, the superoxide dismutase was unable to counteract that process, stimulating CAT and GPx activities. The Integrated Biomarker Response showed that the Tecolutla river had higher values in the upper reaches than the Tuxpan river, showing a decreasing downstream gradient in both seasons. In the Tuxpan river, during the Dry season, the IBR score showed an increasing downstream gradient. During the Northern wind season, the IBR was higher in the upper reaches of both rivers, possibly due to the increased materials transported by runoff from the catchment, which includes a complex mixture of xenobiotics that affects the health of the sentinel species and aquatic biota in general. Based on our results, *Physella acuta* is proposed as sentinel species.

## Introduction

Freshwater ecosystems may well be the most endangered ecosystems in the world (Dudgeon et al., 2005). Water has been the vehicle through which humanity has discharged waste for a long time. In that sense, the rivers, and lakes, among other water bodies, may contain an infinite amount of contaminant compounds that most likely would be impossible to quantify. Therefore, evaluating the toxic effects of water on the organisms living in water bodies turns out to be more effective than measuring the various contaminants in the water. If these pollutants exert adverse effects on the biota, then they are causing damage to ecosystems.

Habitats and species living in freshwater are generally more prone to extinction than terrestrial or marine ones (Tejeda-Vera et al., 2007). The aquatic ecosystem’s health can be severely compromised by various pollutants such as pesticides, heavy metals, hydrocarbons, emerging pollutants, and a complex mixture of many contaminants related to anthropogenic activities (Rhind 2009; Baines et al., 2021). Various chemicals are routinely applied to agricultural lands to control plagues and fertilize the land. The final destinies of many of these pollutants are water bodies. In addition, wastewaters are directly discharged without treatment in streams, rivers, and lakes, mainly. Moreover, all the impacts of human activities and natural events happening in the watershed are reflected in the water bodies. Therefore, aquatic contamination problems are presently more complex, involving chemical mixtures in one or more environmental compartments (e.g., water, sediment, biological tissue/material) (Wepener, 2008).

The exposure or effect of some contaminants can be monitored at various levels such as biochemical, physiological, individual, populations, communities, and ecosystems. Although the population, community, and ecosystem levels are essential, toxic effects are manifested in the first place at the molecular level by impaired biological function. Thus, when the xenobiotics are input into a water body, the pollutants affect individuals in the first place. Therefore, the individual organization level is an appropriate biomonitoring tool for biological early warning systems. Usually, bioassays use mortality as an endpoint; however, in recent years, sub-lethal endpoints have been more sensitive because an organism’s first reaction to stress is physiological in nature (Gerhardt, 1996). Biological early warning responses are fast (within a few hours) and sensitive (at low exposure levels) reactions to pollutant stress beyond homeostasis, causing impairment of the organism health. As long as these responses are compensatory, the disability of the organism is low. Beyond the compensatory range, however, disability increases exponentially (Depledge 1989; Gerhardt, 1996). Specifically, the enzymes usually respond rapidly and sensitively to biologically active pollutants (Fitzpatrick et al., 1997).

Pollutants with redox potential, such as pesticides, heavy metals, medicine metabolites, organic compounds, and their mixtures, among others, can induce the formation of free radicals, which, being oxidants, at high concentrations are agents that are potentially toxic to the cell. Free radical can be defined as any chemical species that contains unpaired electrons in their outer orbit and thus can react virtually with all cell components (Ahmed, 2005). Specifically, oxyradicals or Reactive Oxygen Species (ROS) such as superoxide (O_2_^−^) and hydroxyl (OH^−^) are continually produced as undesirable toxic by-products of normal metabolism from various endogenous processes (Livingstone, 2003), as well as hydrogen peroxide (H_2_O_2_), that is non-radical but is the precursor of hydroxyl through the Fenton reaction (Hermes-Lima et al., 1998), which is one of the most dangerous ROS.

Oxidative stress is an expression used to describe various deleterious processes resulting from an imbalance between the excessive formation of ROS and limited antioxidant defenses (Turrens 2003; Arauz et al., 2016). As a consequence of this imbalance, an oxidative event occurs in which biomolecules are oxidized by ROS, the extent of the damage being dependent on their susceptibility and the exposure time. The basic homeostatic condition can change either due to increased ROS production or reduced defenses (Monaghan et al., 2009). The reactive oxygen intermediates (including superoxide and hydroxyl radicals as well as hydrogen peroxide) can cause direct cellular injury over critical biological molecules, notably DNA, proteins, and lipids, which can all be adversely affected by ROS. Furthermore, the reaction of ROS with these macromolecules generates additional ROS, setting in train a cascade of damage if left unchecked (Monaghan et al., 2009). Lipid peroxidation is a well-established mechanism of cellular injury in both plants and animals and is used as an indicator of oxidative stress in cells and tissues (El-Beltagi & Mohamed, 2013).

Aerobic organisms also synthesize numerous antioxidant enzymes and other proteins to minimize oxidative damage (Hermes-Lima et al., 1998; Livingstone, 2003). Therefore, oxidative stress cannot be inferred simply by measuring just one side of the antioxidant system’s delicate balance between ROS generation and damage limitation by the antioxidant system (Monaghan et al., 2009). Various environmental pollutants induce antioxidant defense enzymes under pro-oxidant conditions (Bebianno et al., 2005). Perhaps the best known of these enzymes is superoxide dismutase. The role of superoxide dismutase (SOD) is to catalyze the dismutation of superoxide radical (O_2_^−^) to hydrogen peroxide (H_2_O_2_) (Murawski et al., 2007). SOD is the most important enzyme because it is found in virtually all aerobic organisms (Wu et al., 2017). The most commonly used antioxidant enzyme biomarkers include catalase (CAT) and glutathione peroxidase (GPx). CAT detoxifies H_2_O_2_ to H_2_O and O_2_, while GPx detoxifies organic peroxides. CAT is an enzyme with high biological relevance because it reduces the concentration of peroxide, a precursor of OH^−^, a highly reactive toxic form of ROS (Vlahogianni et al., 2007). All of these three enzymes are indicators of oxidative stress in cells (Byeon et al., 2021).

Chemical analyses of sentinel species tissues have been used worldwide as a biomarker. Freshwater invertebrates are commonly used in biological monitoring (Sedeño-Díaz et al., 2012; Madrigal-Bujaidar et al., 2017); however, research on biomarkers at the biochemical level is still at the early stage of development (Berra et al., 2004; Rico-Sánchez et al., 2020). Mollusks, mainly bivalves, have been useful as sentinel organisms to assess the effects of pollutants both in marine and freshwater ecosystems (Lima-Melo et al., 2000; Hull et al., 2006; Beltran and Pocsidio, 2010; Musee et al., 2010; Siwela et al., 2010; Klimova et al., 2020). Although freshwater snails perform their whole life cycle in the aquatic environment, they have been barely used as sentinel species. Mainly there are scarce studies on oxidative stress (Bhagat et al., 2016). Freshwater pulmonates (Gastropoda) play an important role in aquatic ecosystems. They transfer energy and materials through the food webs; they are either herbivorous (e.g., Lymnaeidae), feeding mainly upon periphyton, or detritivorous (Lagadic et al., 2007). Several studies agree that using species with great ecological relevance, non-migratory status, sensitivity to contaminants, and being maintained and studied in captivity is the best option to select sentinel species (Grove et al., 2008; Siddig et al., 2016).

Using a sentinel species and determining the effects of a mixture of different pollutants by assessing a combination of biomarkers (e.g., oxidative stress) in laboratory exposures ensures that all aspects of the biochemical effects of xenobiotics exposure are being assessed (López-López et al., 2011). Furthermore, the application of indices capable of integrating the effects of different biomarkers allows for a more holistic view respect to the individual measurements of each biomarker. Such is the case of the Integrated Biomarker Response (Beliaeff and Burgeot 2002), which is an index that simplifies the interpretation of the results of the global variations of a battery of biomarkers by calculating the area of star plots whose vertices represent the scores (standardized data) of each biomarker measured, which is proposed by its authors as an index of ecological risk assessment.

In the Atlantic Mexican slope, several rivers are receptors of wastewater from municipalities, industrial activities, and runoff from agricultural fields. In addition, in some of these areas, there are activities related to exploration, extraction, production, and conduction of crude oil and extractive mining of stony material. Such as the case of Tuxpan and Tecolutla rivers in Veracruz State, which receive these impacts. According to National Commission for the Knowledge and Use of Biodiversity of Mexico (CONABIO 2022), inland waters are classified in Hydrological Priority Regions (HPR) based on their conservation status. In this sense, the Tecolutla river basin represents one of these HPRs, while the Tuxpan river basin is not included in any HPR due to its anthropized condition. Thus, the Tecolutla river is considered one of the best-preserved rivers in Veracruz state (Atlantic slope of Mexico).

In this context, we hypothesize that the Tecolutla river should be considered a reference river concerning others in the same region, despite receiving specific impacts.

If we consider a local species as a sentinel species, it should present adaptive responses to oxidative stress that allow it to perpetuate its permanence in the environment. Thus, this study aimed to assess the biological responses to oxidative stress in a freshwater mollusk exerted by potential toxic effects of water from two rivers on the Mexican Atlantic Slope through the measurement of the level of lipid peroxidation and a battery of antioxidant enzymes. In this regard, *Physella acuta* is being proposed as a test sentinel organism. Additionally, the results are contextualized from an ecological point of view. Thus, the bioassay results were associated with water quality characteristics in each study site.

## Material and methods

### Study area

**FIGURE 1:**
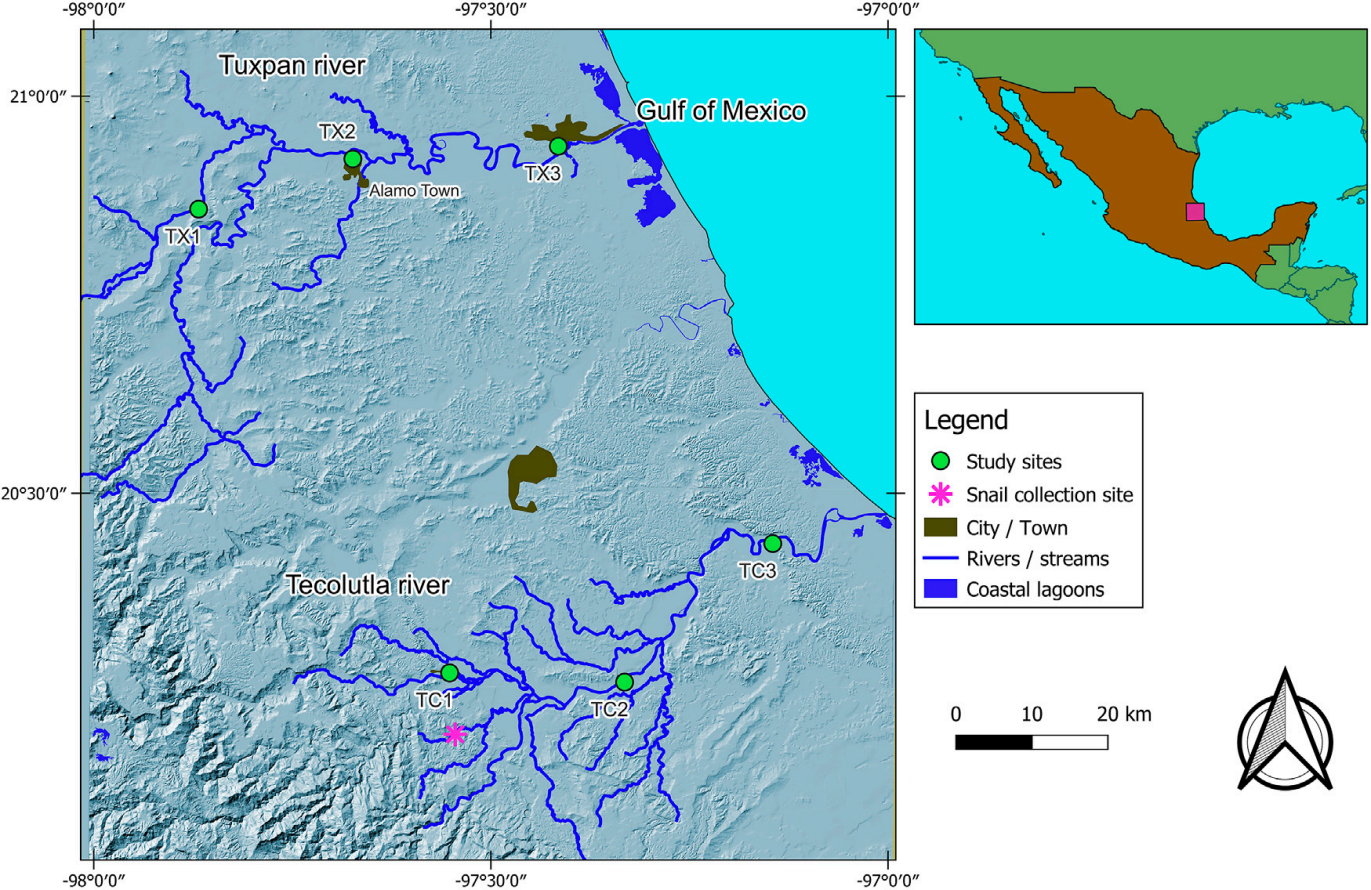
Study area, showing the location of study sites in both rivers and the collection site of sentinel species.

Tuxpan and Tecolutla rivers are two of the most important rivers flowing into the Gulf of Mexico. Both rivers are located in the most Northern tropic humid from the Atlantic Slope (Figure 1). The weather is hot-humid and sub-humid with abundant rainfall throughout the year, particularly in summer (López-López et al., 2009). Also, according to the National Commission for the Knowledge and Use of Biodiversity of Mexico, both rivers are directly influenced by hurricanes and Northern wind seasons. Three study sites along the main river were selected to evaluate the potentially toxic effects of the complex mixture of contaminants present in each river through measurement of lipid peroxidation level and antioxidant system in *Physella acuta*, in two seasons (Northern winds and Dry season). As mentioned above, the Tecolutla river is considered one of the best-preserved rivers on the Atlantic slope of Mexico; we will consider it a reference river. In this sense, we analyze three study sites of each river under the criterion of being at similar altitudes in the three main sections of the river: upper, middle, and lower reaches. The description of the study sites features is as follows:

Tecolutla river. Site 1 (TC1).- This site is in front of the Entabladero town with activities such as fishing, car washing, agriculture, and domestic wastewater discharges; some waste solids are deposited near the littoral. Site 2 (TC2).- Upstream from this site, there are many oil wells and pipeline networks; there are citric farming lands and several river towns upstream, between them the Papantla town. Site 3 (TC3).- This site is subject to tourist activities, traffic of outboard motor boats, and municipal wastewater discharges.

Tuxpan river. Site 1 (TX1).- This site is featured by stony mining material, and during the study, a bridge was constructed at this study site. Site 2 (TX2).- This site is downstream of Alamo town and is subject to stony mining material. Surrounding places are featured by citric farming, and therefore, there is the runoff of different agrochemicals. Site 3 (TX3).- This site is the most downstream and is located near the Tuxpan city, whit a population of over 150,000 inhabitants; it receives impacts from the upper reaches, including citric farming, crude oil operations, and domestic wastewater discharges.

### Field work

At each study site, water samples were collected in the Northern wind season 2020 and Dry season 2021 and transported at 4°C to the laboratory. An additional water sample was collected at each study site to evaluate the physicochemical features. Also, these water samples were transported to the laboratory in the same conditions. Water temperature, conductivity, pH, and dissolved oxygen were measured *in situ* using a Quanta^®^ multiprobe device.

### Water quality

Water samples were tested for sulfates (mgL^−1^), nitrates (mgL^−1^), ammonia (mgL^−1^), color (Unit Pt-Co), O-Phosphates (mgL^−1^), and total phosphorus (mgL^−1^), which were quantified with a Hach DRL 2500 spectrophotometer. Water hardness was measured by titration with EDTA, while biochemical oxygen demand (BOD_5_), total and fecal coliforms, chloride, and alkalinity were analyzed following APHA (2005).

### Test organisms

The bioassays were conducted on the freshwater snail *Physella acuta* (Draparnaud, 1805) with a mean shell length of 9 ± 2 mm. This freshwater snail is an inhabitant of both rivers under study (López-López et al., 2009). This species has a wide distribution in both rivers (Tecolutla and Tuxpam), giving rise to analyze and comparing their biomarker responses in two rivers along a gradient of environmental conditions. We collected samples of these organisms at El Arenal site (a pristine site in the Tecolutla river, Figure 1) and were transferred to the laboratory to be cultivated in dechlorinated tap water. The second generation was considered free of contaminants. Before the exposure test, the sentinel organisms were acclimated. Lots of 48 snails were maintained in six glass aquaria, every containing 20 L of water with a total hardness of 150 mgL^−1^, alkalinity of 31 mgL^−1^ as CaCO_3_, and pH of 7.5 ± 0.5. Water was kept oxygen-saturated by aeration, and its temperature was maintained at ambient laboratory temperature (22 ± 2°C) with a photoperiod of 12:12 light/dark hours. The snails were fed with Warley^®^ commercial fish food. Through bioassay, interference by seasonal variables such as temperature, light, food availability, or reproductive period was avoided.

### Exposure test

Duplicate groups of 24 randomly chosen snails were exposed to TC1, TC2, TC3, TX1, TX2, and TX3 water for 96 h under static conditions (EPA, 1993). During the exposure, the snails were not fed. No mortality was observed in any of the treatments. Due to the small size of these snails, the whole body was used to prepare four pools of three snails from each group to obtain sufficient tissue for all biochemical assays. All biochemical assays were performed in duplicate. A 10% homogenate with chilled potassium phosphate buffer (0.1 M, pH 7.4) was performed with the first two pools of three snails, using a motor-driven homogenizer (Biospecific products Inc. Model 398, United States), to determine CAT and LPO. The second pool of three snails was homogenized with 50 mM potassium phosphate buffer (pH 7.4) to obtain 10% homogenate to determine SOD activity. The last pool was used to prepare another 10% homogenate with 75 mM potassium phosphate buffer (pH 7.0) to quantify GPx activity.

### Biochemical assays

All biomarker analyses were conducted using a spectrophotometer HACH DRL 2500. Superoxide dismutase activity was measured according to the method of Sun et al. (1988) with minor modifications. Standard SOD solutions (0–200 ng/tube) in isotonic saline and the SOD assay reagent containing xanthine, EDTA, Na_2_CO_3_, and nitrobluetetrazolium were made. The reaction was started by adding xanthine oxidase and allowed for 30 min. The activity was determined spectrophotometrically at 546 nm. The amount of bovine liver SOD that inhibited NBT reduction by 50% was defined to be one unit of enzyme activity.

CAT activity was measured through hydrogen peroxide consumption at 480 nm (Cohen et al., 1970). Briefly, 5 ml of cold 6 mM H_2_O_2_ were added to 0.5-ml aliquots of the 10% homogenate pool of snails with chilled potassium phosphate buffer mentioned above. After 3 min, the reaction was stopped by the addition of 1.0 ml of 6 N H_2_SO_4_. The H_2_O_2_ reacted with a standard excess of 0.01 N KMnO_4_, and the residual KMnO_4_ was measured at 480 nm. Catalase activity was calculated as the first-order reaction rate constant of the H_2_O_2_ decomposition (K × 10^2^ min^−1^).

The assay of GPx activity was evaluated by the method of Lawrence and Burk (1976) using cumene hydroperoxide as substrate. Changes in absorbance at 340 nm were recorded at 1 min intervals for 4 min, and the results are expressed as nmol NADPH oxidized min^−1^ mg^−1^ of protein, using a molar extinction coefficient of 6.22 × 106 M^−1^cm^−1^.

Thiobarbituric acid reactive substances (TBARS) were quantified in the 10% tissue homogenate as an indicator of lipid peroxidation, specifically malondialdehyde, as described by Buege and Aust (1978). Briefly, three hundred microliters of 10% homogenate potassium phosphate buffer were added to 700 μL 150 mM Tris–HCl (pH 7.4). The mixture was placed in a cuvette and thermostated at 37°C for 30 min. The mixture was added to 2 ml 0.375% thiobarbituric acid dissolved in 15% trichloroacetic acid and was heated for 45 min in a boiling water bath. After cooling, the mixture was centrifuged at 900 × g for 10 min. The absorbance of each aliquot was measured at 535 nm, and the LPO level was expressed as nmol of TBARS formed per milligram of protein (nmol MDA mg^−1^ prot.) using a molar extinction coefficient of 1.569105 M^−1^ cm^−1^.

### Data and statistical analysis

All values are expressed as mean ± standard error (SE). Significant differences between groups were analyzed by one-way analysis of variance (ANOVA) with Tukey’s multiple comparison test. The significance of the results was ascertained at *p* ≤ 0.05.

The biomarkers data were employed to compute the Integrated Biomarker Response (IBR) in concordance with Beliaeff and Burgeot (2002). IBR for study sites in both seasons was compared to identify significant differences. Thus, general mean (m) and standard deviation (s) were obtained for all study sites for each biomarker. Data for each study site (x) were standardized as follows: First was obtained Y (Y=(x−m)/s). According to Beliaeff and Burgeot (2002), Z = Y. Since no response-inhibiting biomarkers were used, it was not necessary to obtain the value of –Y, as stated by the authors. The minimum value (Min) for all study sites for each biomarker was obtained. Finally, the score S was computed as S= Z + |Min|, where S ≥ 1 and |Min| is the absolute value of the minimum value. Then, star plots by study site were plotted to have as radius the Si value of the score of the ith biomarker. The IBR represents the area closed by a star plot formed by all Si values. According to Beliaeff and Burgeot (2002), IBR was computed as the summatory of the triangles defined by the Si and its consecutive Si + 1 clockwise scores.

A principal component analysis was performed to detect patterns between study sites based on water quality variables, biomarkers, and integrated biomarker response to include the bioassay in an ecological context.

## Results

### Water quality

Some differences in water quality were found between rivers and study sites. The values of environmental factors assessed in both rivers are shown in Tables 1, 2. The water of the Tuxpan river is harder and warmer than the water of the Tecolutla river; however, significant differences were not found (*p* > 0.05). BOD in both rivers and all study sites during the Dry season was higher than the values obtained in the Northern wind season (*p* = 0.0089). However, in the Tecolutla river, these differences were most evident because values of BOD_5_ in the Dry season were 2–4 fold higher than the Tuxpan river in the same period. Nitrate concentrations in the Tecolutla river were significantly higher than the Tuxpan river in both study periods (*p* = 0.003). Conductivity and chlorides in both rivers increased downstream during the Dry season.

**TABLE 1:** Water quality features for Tecolutla river.

Site	TC1		TC2		TC3	
	Northern wind season 2007	Dry season 2008	Northern wind season 2007	Dry season 2008	Northern wind season 2007	Dry season 2008
DO (mgL^−1^)	9.51	8.83	9.8	9.25	8.61	11.01
BOD (mgL^−1^)	0.76	13.03	0.60	13.59	1.44	15.41
Nitrates (mgL^−1^)	1.15	0.9	1.2	1.1	0.55	1.45
Ammonia (mgL^−1^)	0.225	0.215	0.205	0.185	0.250	0.200
O-Phosphates (mgL^−1^)	0.125	0.26	0.12	0.31	0.05	0.155
Total Phosporus (mgL^−1^)	0.7	0.5	0.6	0.38	0.635	0.31
Hardness (mgL^−1^)	71	99	29	110	217	500
Color (Unit Pt-Co)	1	1	1	5	1	1
Total Coliforms (MPN/100 ml)	1600	4	500	9	1600	1600
Fecal Coliforms (MPN/100 ml)	900	4	11	7	29	1600
Sulfates (mgL^−1^)	15.5	18	11.5	27	15	20.5
Alkalinity (mgL^−1^)	88.4	173.825	86.4	199.38	106.4	191.71
Chloride (mgL^−1^)	9.99	76.07	7.19	252.90	2343.07	10566.72
Conductivity (μs cm^−1^)	224	240	221	258	531	11180
pH	8.47	8.67	8.54	8.76	8.33	8.81
Water temperature (°C)	19.56	20.53	22.65	20.67	22.09	20.09

**TABLE 2:** Water quality features for Tuxpan river.

Site	TX1		TX2		TX3	
Tuxpan river	Northern wind season 2007	Dry season 2008	Northern wind season 2007	Dry season 2008	Northern wind season 2007	Dry season 2008
DO (mgL^−1^)	10.71	9.08	8.01	7.15	9.18	8.38
BOD (mgL^−1^)	0.75	2.24	1.14	2.24	2.58	7.62
Nitrates (mgL^−1^)	0.4	0.35	0.45	0.6	0.45	0.6
Ammonia (mgL^−1^)	0.18	0.19	0.175	0.19	0.14	0.215
O-Phosphates (mgL^−1^)	0.08	0.185	0.125	0.18	0.115	0.08
Total Phosporus (mgL^−1^)	0.4	0.26	0.48	0.295	0.41	0.235
Hardness (mgL^−1^)	100	143	122	185	579	500
Color (unit Pt-Co)	0	40	11	9	12	36
Total Coliforms (MPN/100 ml)	300	1600	220	50	1600	23
Fecal Coliforms (MPN/100 ml)	17	1600	170	23	110	6
Sulfates (mgL^−1^)	15	18	20	26	20	27.5
Alkalinity (mgL^−1^)	107.6	120.8	137.6	114.8	158.4	147.6
Chloride (mgL^−1^)	91.97	10.39	107.96	11.99	6777.89	3927.15
Conductivity (μs cm^−1^)	270	280	317	290	200	3160
pH	8.82	9.28	8.66	9.00	8.68	8.93
Water temperature (°C)	21.21	27.88	20.93	28.44	21.44	27.72

### Biochemical assays

**FIGURE 2:**
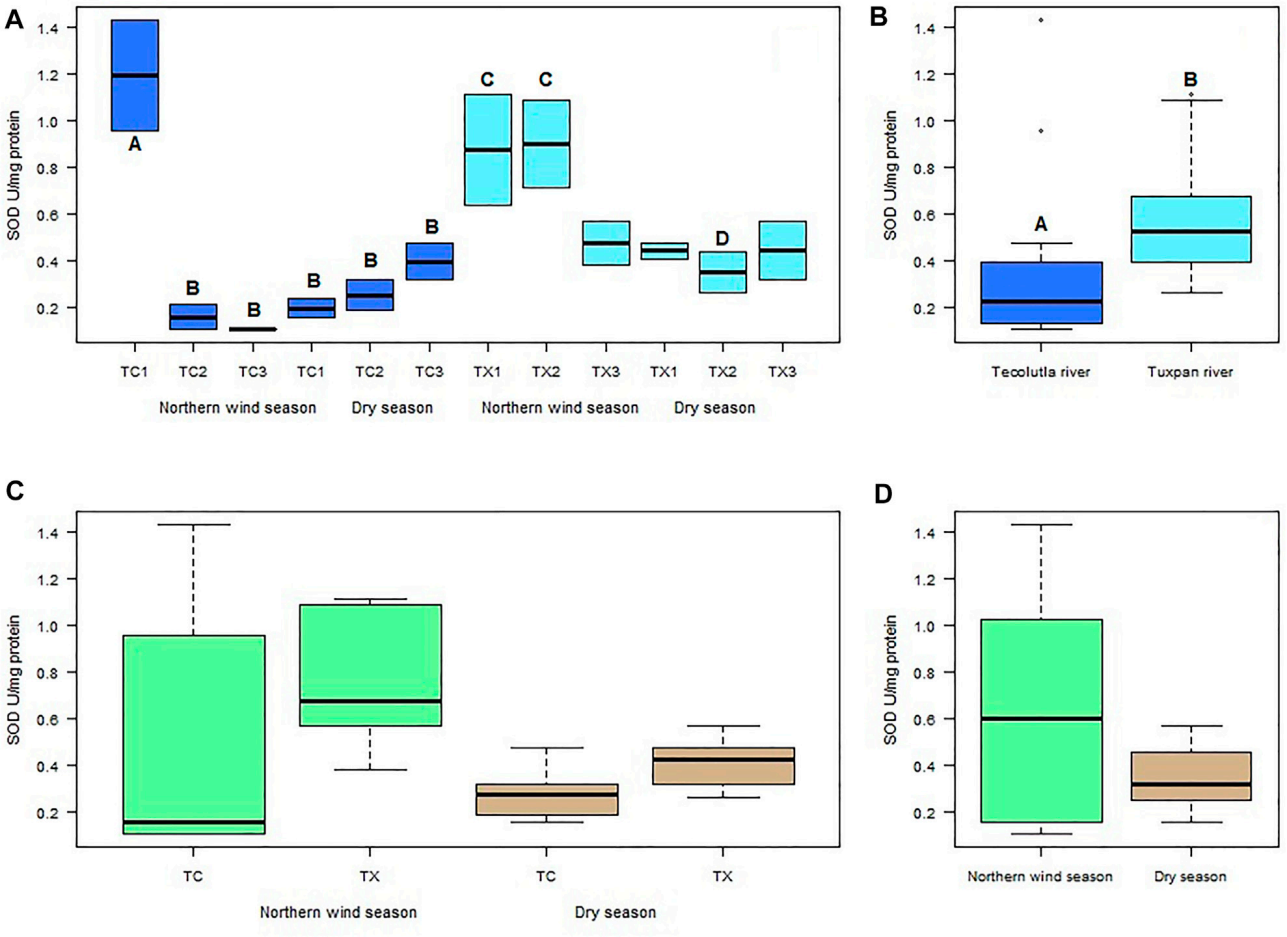
Boxplot for SOD activity in *Physella acuta*. **(A)** SOD activity by study site and season; **(B)** SOD activity by river; **(C)** SOD activity by river and season; and **(D)** SOD activity by season. N = 24 by study site, *n* = 72 by season, and *n* = 144 by river (A ≠ B, C ≠ D).

Spatial and temporal differences were found in mollusks exposed to water of the Tecolutla and Tuxpan rivers. In the Tecolutla river, the highest mean value of SOD activity measured in *P. acuta* was observed in organisms exposure to water of study site TC1 (1.19 U mg^−1^ Protein) during Northern winds season; this activity was significantly higher than those of other study sites in both seasons (*p* < 0.05). The lowest mean value of SOD activity was found in organisms exposed to water of study site TC3 (0.106 U mg^−1^ Protein), also in the Northern wind season (Figure 2A). In the Dry season, a gradient with increasing values was observed from the study site TC1 to TC3, that is, from the upper to the lower reaches of the Tecolutla river.

In the Tuxpan river, the highest values of SOD activity were always in the Northern wind season for each study site; whereas in the Dry season, the mean values of SOD activity were lowest in TX2 site (Figure 2B); however, significant differences were not detected between study sites in the Dry season. Between seasons, TX1 and TX2 in the Northern wind season were different to TX2 in the Dry season (*p* < 0.05) (Figure 2A).

Globally, the exposure of test organisms to Tuxpan river water significantly (*p* < 0.05) promoted more SOD activity than those exposed to water from the Tecolutla river (Figure 2B). Seasonally, the Tuxpan river exhibit more SOD activity than Tecolutla river (which showed an outlier). In the Dry season, the Tuxpan river exerted more SOD activity than the Tecolutla river in both seasons (Figure 2C); likewise, the Norther wind season exhibited higher SOD activity than that observed in the Dry season (Figure 2D).

**FIGURE 3:**
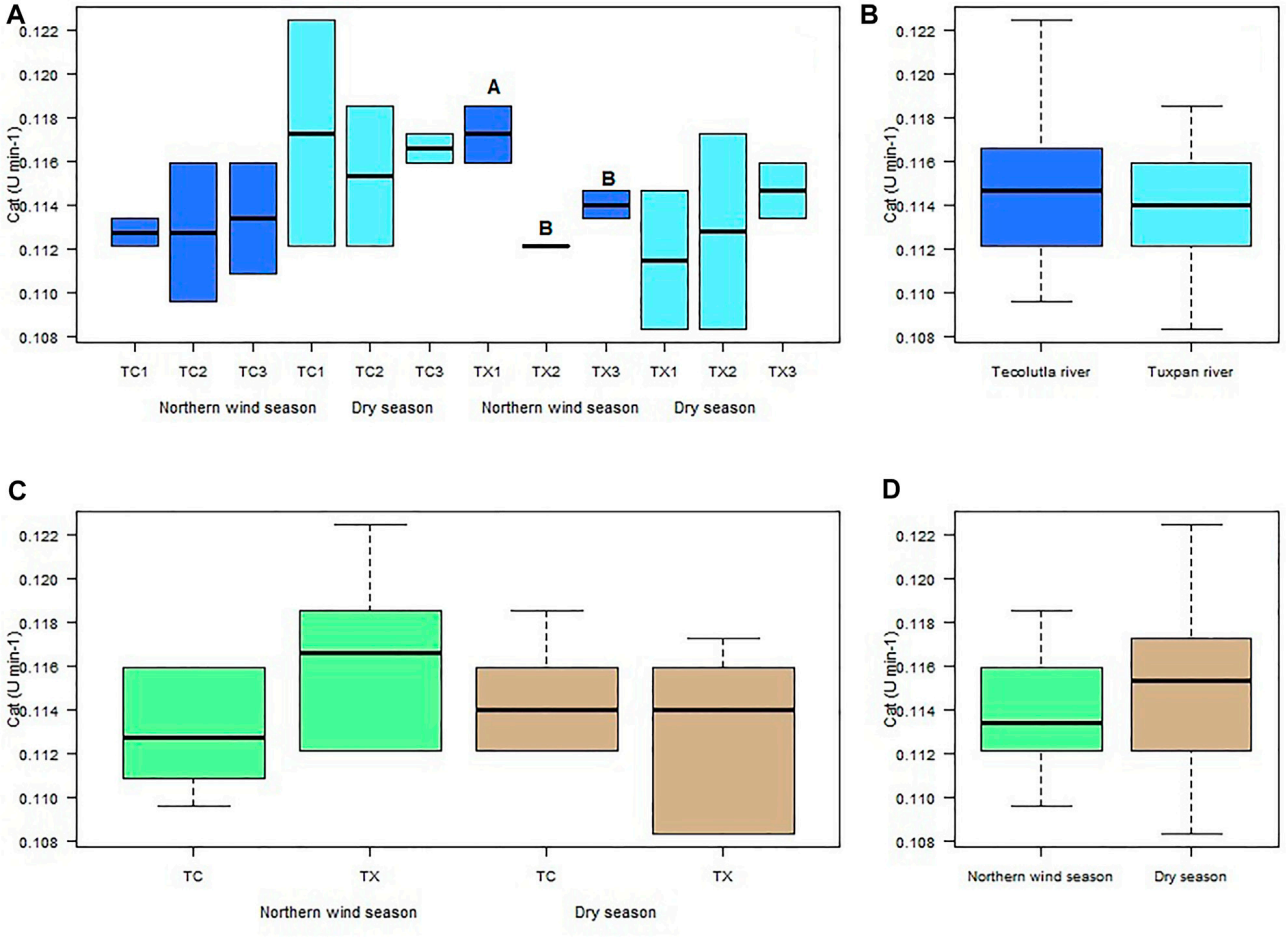
Boxplots for CAT activity in *Physella acuta*. **(A)** CAT activity by study site and season; **(B)** CAT activity by river; **(C)** CAT activity by river and season; and **(D)** CAT activity by season. N = 24 by study site, *n* = 72 by season, and *n* = 144 by river (A ≠ B).

Despite CAT activity in organisms exposed to water from study sites of the Tecolutla river in both seasons, does not differ significantly between them (*p* > 0.05), the CAT activity in organisms exposed to water from TC1 during the Dry season showed higher values than during the Northern wind season (Figure 3A). In the Tuxpan river, the highest CAT activity was in TX1 in the Northern wind season, which was different significantly with TX2 and TX3 in the same season (*p* < 0.05). In the Dry season, there were no significant differences between study sites (*p* > 0.05); however, a slight increment from TX1 to TX3 can be observed (upstream to downstream) (Figure 3A).

The CAT activity of *Physella acuta* exposed to water of the two rivers in both seasons did not was different significantly (*p* > 0.05) (Figures 3B–D). However, the Tecolutla river shows slightly higher CAT activity than the Tuxpan river (Figure 3B). In the Northern wind season, the Tuxpan river showed slightly higher values of CAT than the Tecolutla river (Figure 3C). Finally, Tecolutla and Tuxpan rivers showed higher CAT activity in the Dry season than in the Northern wind season (Figure 3D).

**FIGURE 4:**
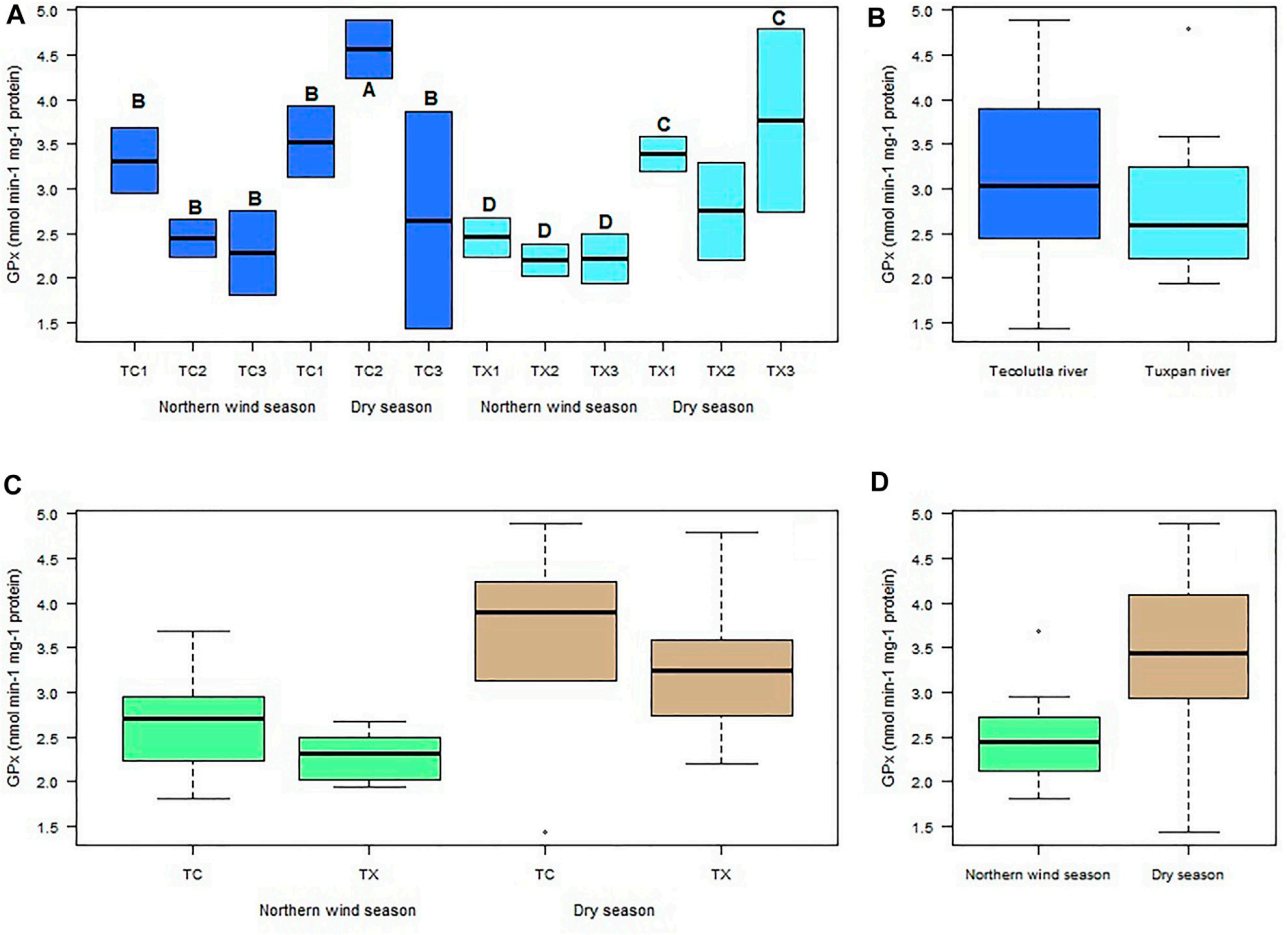
Boxplots for GPx activity in *Physella acuta*. **(A)** GPx activity by study site and season; **(B)** GPx activity by river; **(C)** GPx activity by river and season; and **(D)** GPx activity by season. N = 24 by study site, *n* = 72 by season, and *n* = 144 by river (A ≠ B, C ≠ D).

The highest GPx activity in mollusks exposed to water of Tecolutla river was detected in TC2 in the Dry season, which showed significant differences (*p* < 0.05) with all study sites during the Northern wind season and with TC1 and TC3 in the Dry season. In the Tuxpan river, significant differences (*p* < 0.05) were found in TX1 and TX3 in Dry season, in relation to all study sites in the Northern wind season. However, there were no significant differences between study sites in each season (Figure 4A). Despite no significant differences between rivers, the Tecolutla river exhibited higher GPx activity than the Tuxpan river (Figure 4B). In a global sense, GPx activity was higher in the Dry season than in the Northern wind season (Figures 4C,D).

**FIGURE 5:**
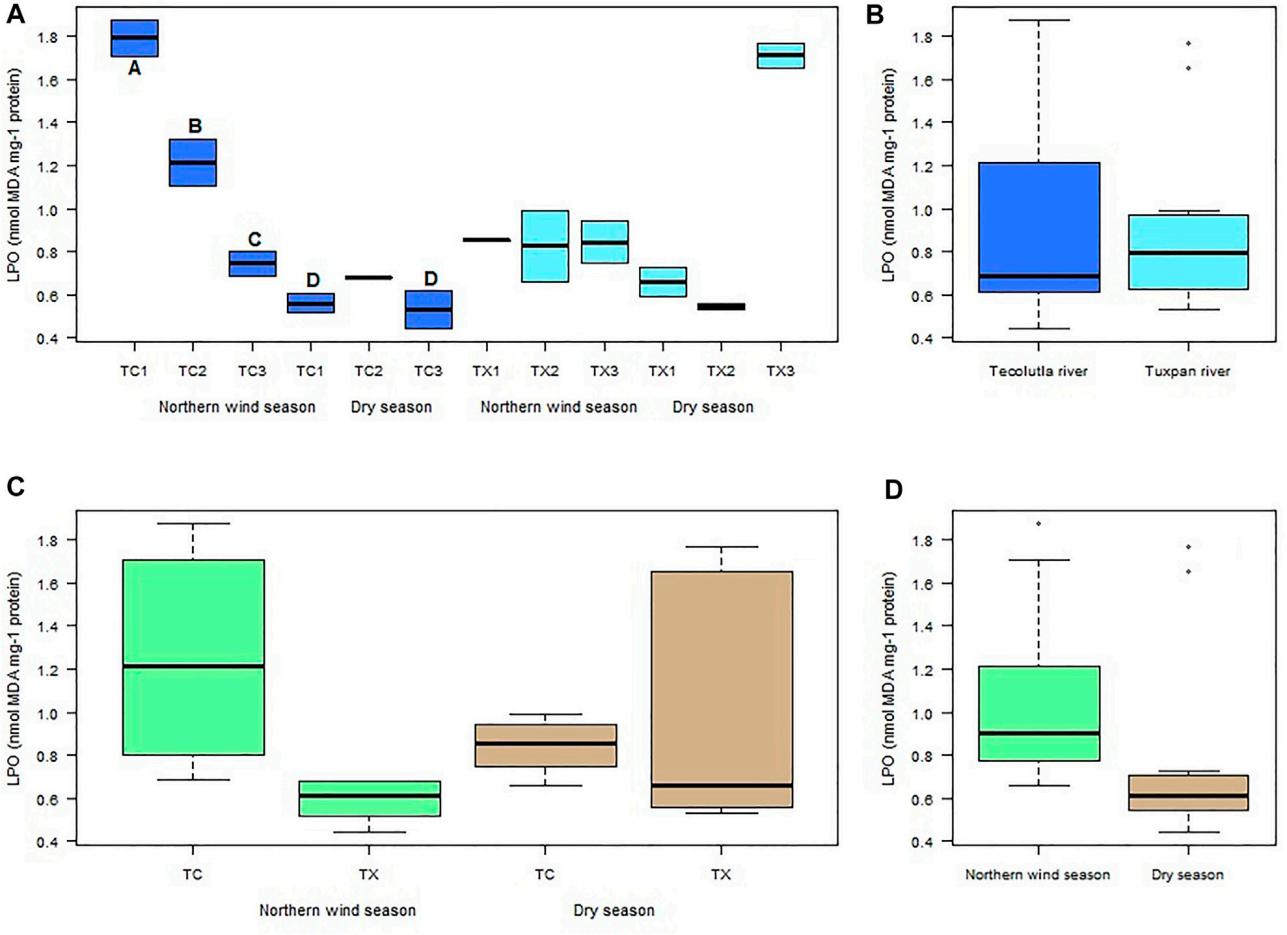
Boxplots of LPO levels in *Physella acuta*. **(A)** LPO level by study site and season; **(B)** LPO level by river; **(C)** LPO level by river and season; and **(D)** LPO level by season. N = 24 by study site, *n* = 72 by season, and *n* = 144 by river (A ≠ B ≠ C ≠ D).

LPO in the Tecolutla river was consistently higher in the Northern wind season than Dry season in all study sites. A gradient in LPO values from TC1 to TC3 was observed in the Northern wind season, with significant differences between study sites (*p* < 0.05). LPO values in the Dry season did not show significant differences between study sites. However, TC1 and TC3 in this season were significantly different from study sites in the Northern wind season (*p* < 0.05) (Figure 5A). There were no significant differences in LPO values between Tecolutla and Tuxpan rivers (*p* > 0.05) (Figure 5B). Likewise, there were no significant differences between seasons (Figure 5C). Globally, there were observed higher values of LPO in Norther wind season than in Dry season (*p* > 0.05, Figure 5D).

### Integrated biomarker response

**FIGURE 6:**
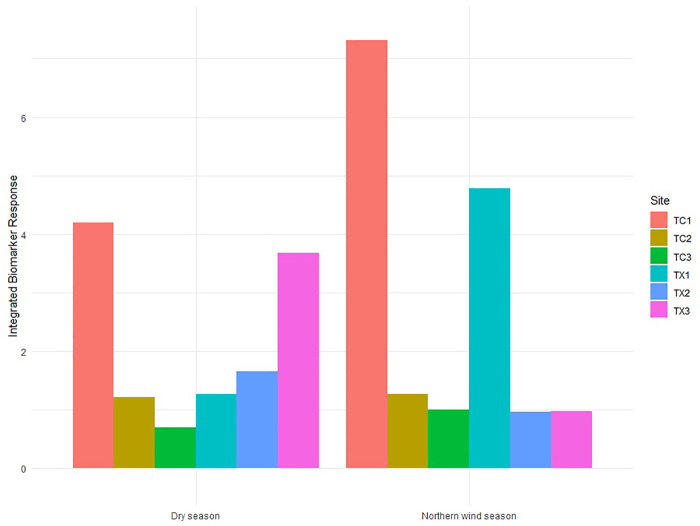
Integrated Biomarker Response scores in *Physella acuta* by season and study site based on the four biomarkers measured (*n*= 24 by study site).

Scores of IBR showed temporal and spatial differences. During the Northern wind season, the IBR score was higher in TC1 and TX1 (the study sites in the upstream reach of each river) than those downstream study sites in both rivers. The IBR scores for study sites 2 and 3 for each river did not show differences. Tecolutla River consistently exhibited a gradient in IBR scores from upstream to downstream reaches in both seasons, with the maximum IBR score in TC1 and the minimum in TC3. In the Tuxpan river, during the Dry season, IBR scores increase from upstream to downstream (Figure 6), indicating the increasing pollution downstream.

### Ecological context

**FIGURE 7:**
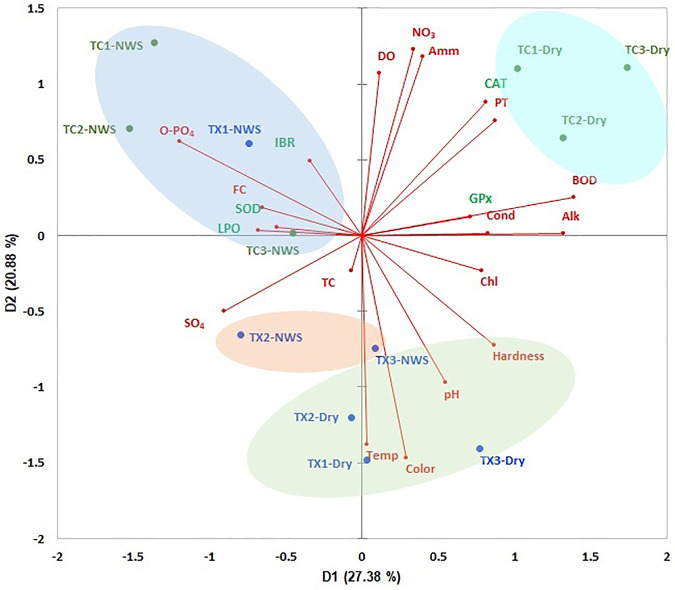
ACP biplot of environmental variables of both rivers, and biomarkers, and IBR in *Physella acuta*. Ovals indicate the arrangement of study sites (NWS= Northern wind season).

The PCA showed an accumulated variance of 48.26% in the first two components (Figure 7). The study sites of each river were ordered based on environmental variables, LPO level, antioxidant enzymatic responses, and IBR. Study sites of the Tecolutla river during the Northern wind season showed a high correlation with the highest values of O-Phosphates, fecal coliforms, SOD, LPO, and IBR; the study site TX1 also shows a relationship with the same parameters. As mentioned above, IBR scores in the two rivers were high in the upstream study sites (for the Tuxpan river, only during the Northern wind season). During the Dry season, study sites of the Tecolutla river were influenced mainly by BOD_5_, PT, NO_3_, and Ammonia, which can be related to the presence of wastewater discharges from the activities previously mentioned. However, these study sites also were associated with high values of DO. Tuxpan river during the Dry season showed alkaline water (highest values of pH), high values of hardness, and water temperature. According to SOD, LPO vectors, and IBR, these sites during the Dry season showed the least oxidative stress. During the Northern wind season, study sites TX2 and TX3 showed high values of sulfates.

## Discussion

Oxidative stress occurs when the antioxidants (enzymatic and non-enzymatic systems) cannot thoroughly neutralize the ROS produced (Halliwell, 2007). In our study, TBARS measurements indicate that lipid peroxidation occurs in snails exposed to water of different sites of both rivers at different intensities. That is, the mixture of xenobiotics dissolved in water potentially induces ROS formation on sentinel species. When an oxidative process begins in an organism, biochemically, the first and the most effective antioxidant enzyme that acts against free radicals formation process is SOD (Ighodaro and Akinloye, 2018; Yang et al., 2019). The highest value of SOD was obtained in TC1 in the Northern wind season, just when the LPO was also highest. The significant increase in SOD values may be a response of the first defense system to ROS induced by xenobiotics in the tested water, as Ighodaro and Akinloye (2018) indicated. SOD in TC1 was 11.2 fold higher in the Northern wind season than TC3 in the same period. This significant increment is directly related to the highest level of LPO. In concordance to Carvalho et al. (2012), the increase of the SOD activity leads to a rise in H_2_O_2_ production that cannot be compensated by the CAT and GPx, thereby inducing oxidative stress and stimulating lipid peroxidation. The fact that SOD activity and LPO showed both elevated values suggest that there is an excess of superoxide anion. Vlahogianni et al. (2007) found in the mussel *Mytilus galloprovincialis* in coastal areas of Saronikos Gulf of Greece that SOD increased when LPO also showed a high level.

The role of SOD is to accelerate the dismutation of the toxic superoxide radical (O_2_^−^) produced during oxidative processes to hydrogen peroxide and molecular oxygen (Murawski et al., 2007). Likewise, CAT plays a key role in enzymatic oxidant defense, converting hydrogen peroxide into oxygen and water (Bhagat et al., 2016). However, the excess concentration of superoxide radical itself directly affects the activity of CAT and peroxidases (Kehrer 2000). In the Tecolutla river, during the Northern wind season, the excess of superoxide radical (measured as TBARS) stimulated the increase of SOD activity (TC1). Nevertheless, CAT activity values were low. While SOD acts reducing the superoxide radical and generating hydrogen peroxide, the low values of CAT were not able to stop the process of lipid peroxidation by reduction of peroxide. Kehrer (2000) indicates that it is possible to form radical hydroxyl through the Fenton reaction since the peroxide is the primary source. Thus, our results concordance with Pandey et al. (2003), who found that SOD activity in all the fish *Wallago attu* was high, whereas the CAT activity was found to be low in all tissues. Inhibition of CAT has been suggested as a transitory response to acute pollution (Wang et al., 2014; Bhagat et al., 2016). Vasseur and Cossu-Leguille (2003) mentioned that induction of an antioxidant system traduces an adaptive reaction to a perturbed environment. In contrast, an inhibition is predictive of cell damage and reflects the toxicity of bioavailable pollution.

In this study, when SOD, CAT, and GPx activities were high, the lowest values of LPO levels were recorded (Tecolutla river during the Dry season). The coupled antioxidant system SOD, CAT, and GPx can carry on a ROS detoxification process, as indicated by Bhagat et al. (2016). These are interdependent biomarkers that can be interpreted as an adaptive response.

LPO levels were high in TX3 during the Dry season, and the SOD activity was low. However, CAT and GPx exhibited high activity, indicating that the second and third levels of the antioxidant system stop the lipid peroxidation process. These results are in concordance with Siwela et al. (2010), who founds significantly low activity of SOD and increased level of LPO in tissues of the *Lymnaea natalensis*, a freshwater snail.

In our study, SOD activity in TX1 and TX2 in both seasons was enough to counteract the LPO effects. However, in TX3 in the Dry season, LPO exceeded the capacity of SOD. Hence CAT and GPx acted to counteract the lipid peroxidation process. TX3 is located in the lowest reaches of the Tuxpan river, where runoff is integrated and is the most polluted zone.

In order to diagnose and compare the health status of snails facing oxidative stress (due to exposure to a mixture of xenobiotics potentially generating ROS), the IBR was applied to the six study sites and four biomarkers. Wang et al., 2010 called the IBR a “stress index”. In our case, the IBR could be called as “*Integrated Oxidative Stress Response*” since we evaluated the LPO and some antioxidant enzymes.

Lehtonen et al. (2006) used the IBR to assess the pollution status of *Mytilus edulis* and *Macoma balthica* collected from the southern coast of Finland. They found through the IBR, the most contaminated site, as well as a gradient of IBR values. In this study, the IBR score showed important differences among study sites and periods experienced by *P. acuta* when exposed to water of both rivers. Since no snail died during the experimental period, the IBR reflects the homeostatic mechanism by which the snails regulate their metabolism when facing a stressful condition. IBR indicated that the water of TC1 was the potentially higher risk, both in the Northern wind and the Dry seasons. Likewise, IBR values exhibited a decreasing gradient in both seasons from upper to lower reaches.

On the other hand, the Tuxpan river exhibited the highest value of IBR in TX1 during the Northern wind season, while TX2 and TX3 in the same period showed the lowest values of IBR in both seasons. Therefore, the Northern wind season affects the upper reaches of the Tuxpan River, possibly due to increased runoff and transport of materials from the catchment, which represents an increase in stressful conditions and increased risk to the sentinel species and the biota in general in that portion of the river. In contrast to the Tecolutla river, IBR in the Tuxpan river shows an increasing gradient from upper to lower reaches during the Dry season. Tecolutla River consistently exhibited a gradient in IBR scores from upstream to downstream in both seasons, with the maximum IBR score in TC1 and the minimum in TC3. These results show that the Tecolutla river presents stressful conditions in its upper reaches, even though it should present pristine conditions since it has been considered the best-preserved river according to CONABIO (2022).

The ecological context shows that even though fecal coliforms alone do not exert LPO on mollusks, they are indicators of municipal wastewaters. Wastewaters represent a complex mixture of xenobiotics that can include organic matter, which, in this case, is represented by a high concentration of phosphorus, nitrogen, and BOD_5_. Likewise, such a complex mixture may also have hydrocarbons (Ozaki et al., 2019), pharmaceuticals (Scholz et al., 2009), and heavy metals (Du et al., 2020), which are promoters of oxidative stress. In the Dry season in the Tecolutla river, high nitrates and conductivity values seem to promote the GPx activity. GPx is the most critical peroxidase for detoxifying hydroperoxides (El-Shenawy et al., 2012). While conductivity is an integrative parameter of all dissolved solids in water, among them compounds such as those mentioned above; then, the higher values of GPx may be due to all masked compounds in water conductivity. In addition, *Physella acuta* is considered a freshwater species, and increasing salinity can provoke oxidative stress. Nitrates, ammonia, phosphorus, and coliforms can be derived from wastewater discharges and agricultural runoff. The complex mixture of xenobiotics in municipal wastewater discharges contains pro-oxidant agents that generate ROS in *P. acuta*.

Similarly, the nutrients from agricultural runoff imply the presence of other agrochemicals, their metabolites, and by-products, which also forming potentially ROS. These results are in concordance with Castañeda-Chávez and Lango-Reynoso (2021). They found that the highest values of some pesticides in the Jamapa river, Veracruz, Mexico, were recorded in the Northern wind season. Since the highest values of IBR were observed in the Northern wind season, this period is considered to provoke more biological damage to sentinel species and, therefore, to aquatic biota in general, mainly in the Tuxpan river. The wastewater discharges, the extraction of stone materials and hydrocarbons, and the agricultural areas result in high concentrations of nutrients and pro-oxidants, affecting the quality of both rivers. Therefore, the Tecolutla river cannot be considered to be in a good conservation condition, and its upper reaches are even more affected than the upper portion of the Tuxpan River.

The oxidative damage and antioxidant response to xenobiotics have been widely investigated in freshwater organisms, mainly in fish (Livingstone, 2003; Sahan et al., 2010; Trujillo-Jiménez et al., 2011) and some invertebrates (Berra et al., 2004; Guidi et al., 2010) but in freshwater snails are scarce (Siwela et al., 2010; Khalil 2015). *Physella acuta* has been shown to be an excellent sentinel organism since it is very sensitive to water quality changes and its antioxidant system responses were fast and sensitive, as indicated in Gerhardt, 1996, being able to display adaptive responses. Likewise, the battery of biomarkers related to oxidative stress and antioxidant enzymatic response is an efficient early warning sign to identify oxidative stress. Although TC1 was the study site in the most upstream portion of the Tecolutla river, it showed signs of pollution affecting the health of *P. acuta*, because the antioxidant enzyme system was unable to stop the oxidative process. These results indicate that the Tecolutla river is already showing deterioration in its upper reaches. Therefore, we propose to freshwater snail *P. acuta* as a sentinel organism for studies of oxidative stress. The IBR was a valuable tool for quantifying oxidative damage and its effect on antioxidant enzymes.

## Data Availability

The raw data supporting the conclusions of this article will be made available by the authors, without undue reservation.
